# Incidence and related risk factors of radiographic knee osteoarthritis: a population-based longitudinal study in China

**DOI:** 10.1186/s13018-021-02577-1

**Published:** 2021-07-31

**Authors:** Liyi Zhang, Chutong Lin, Qiang Liu, Jiaxiang Gao, Yunfei Hou, Jianhao Lin

**Affiliations:** 1 Arthritis Clinic and Research Center, Peking University People’s Hospital, Peking University, No. 11 Xizhimen South Road, Xicheng District, Beijing, 100044 China; 2 Arthritis Institute, Peking University, Beijing, China; 3 Department of Thoracic Surgery, Peking University Third Hospital, Peking University, Beijing, China

**Keywords:** Knee osteoarthritis, Incidence, Risk factors, Radiograph

## Abstract

**Objective:**

To explore the incidence and risk factors for radiographic knee osteoarthritis (ROA) in a suburban area of China.

**Methods:**

Shunyi Osteoarthritis Study was a population-based, longitudinal study of knee osteoarthritis in Shunyi, a suburban area of Beijing, China. A total of 1295 residents aged over 50 years were recruited with fully informed by randomized cluster sampling and were followed up 3 years later. At the time of baseline and follow-up visits, participants completed a home interview questionnaire and received a clinical examination including height, weight, range of motion (ROM), chair stand test, 50-foot walk test, and weight-bearing posterior-anterior semi-flexed view of radiographs at tibiofemoral joints. The incident ROA for a knee was defined if its KL grade was no more than grade 1 at baseline visit and no less than grade 2 at the follow-up visit. A patient without ROA in both knees at the baseline visit and with ROA in at least one knee at the follow-up visit was viewed as an incident case of ROA in patient level. Generalized linear model and generalized estimating equation were performed to examine the association between socio-demographic factors, physical function as well as baseline knee joint condition, and incident ROA in patient and knee level.

**Results:**

A total of 1295 residents were recruited at baseline in 2014, and 962 (74.3%) residents were followed in 2017. The annual cumulative incidence of ROA was 3.6% at knee level and 5.7% at patient level. Older age (per year, adjusted odds ratio (OR) = 1.079; 95% confidence interval (CI), 1.042-1.117), overweight (adjusted OR = 2.086; 95% CI, 1.286-3.385), female (adjusted OR = 1.756; 95% CI, 1.074-2.877), less ROM (per degree, adjusted OR = 0.952; 95% CI, 0.923-0.983) and Kellgren and Lawrence (KL) grade 1 at baseline (adjusted OR = 8.527; 95% CI, 5.489-13.246) were risk factors for incident ROA.

**Conclusion:**

The incidence of knee ROA in Chinese suburban area was high. Advanced age, female, overweight, less range of motion, and KL grade 1 at baseline were associated with an increased risk of incident ROA.

## Introduction

Knee osteoarthritis (OA) is one of the most prevailing forms of osteoarthritis, causing pain, stiffness, deformity, and functional disability, which further leads to cardiovascular events and increases all-cause mortality [1–3].

Obesity and aging have long been shown to be strong risk factors for the incidence and progression of knee osteoarthritis. With the aging population and increase prevalence of obesity, this disorder and total knee replacement have become substantially more common in recent decades [4]. According to the 2010 Global Burden of Diseases study, the burden of OA is increasing most rapidly among musculoskeletal disorders in terms of disability-adjusted life years, which will impose new challenges on health system [5]. Early detection and prevention of knee OA is very important. However, there has been a gap between research and clinical practice in the field of prevention of knee OA. Filling this gap has been increasingly emphasized according to the concept of translational orthopedics [6]. Therefore, it is of necessity to understand the epidemiology of knee OA and identify the associated demographic factors.

Although a large number of potential risk factors for incident radiographic knee OA have been studied, most of those studies were performed in European population, and were limited by their cross-sectional design, and moreover some results are conflicting [7–9]. There were a few longitudinal studies on the risk factors of knee OA among Asian peoples, most of which were studied in Korea and Japan [10, 11]. Longitudinal population-based studies in Chinese population are rare, and the incidence and risk factors of OA in this large developing country are still unclear. We aimed to give the latest morbidity data and identify risk factors of knee OA in Chinese population, giving reference to medical policy makers.

The purpose of this population-based longitudinal study was to explore the incidence and risk factors for radiographic knee OA among the Chinese in suburban community.

## Subjects and methods

### Subjects

The Beijing Shunyi osteoarthritis study was a population-based, longitudinal, and prospective study of knee osteoarthritis, which was approved by the Ethics Committee of Peking University People’s Hospital. The study was based on a random cluster sampling method in 2014. Fourteen villages in Shunyi District, Beijing, China, were selected, and every resident who participated in the survey had signed the informed consent document. The inclusion criteria for this study were (1) residence in Shunyi District, Beijing, and (2) age over 50 years. Exclusion criteria: (1) people with rheumatoid arthritis, (2) physically disability, (3) mental retardation, (4) people with advanced malignant tumors or bedridden people, and (5) people recently living or working outside for more than 6 months. And the rejection criteria: (1) people who did not meet the inclusion criteria, (2) people who met the exclusion criteria, (3) people who failed to complete the main records, and (4) people who showed obvious incompatibility in the survey.

At the time of each visit, subjects completed a home interview questionnaire and received a clinical examination including height, weight, range of motion, chair stand test, 50-foot walk test, and weight-bearing posterior-anterior semi-flexed view of radiographs at tibiofemoral joints.

### Assessment of radiographic osteoarthritis

Radiographic osteoarthritis (ROA) was assessed using the Kellgren and Lawrence (KL) criteria (0 = none; 1 = possible osteophytes only; 2 = definite osteophytes and possible joint space narrow (JSN); 3 = moderate osteophytes and/or definite JSN; and 4 = large osteophytes, severe JSN, and/or bony sclerosis) [12]. Radiographic OA was defined as a KL grade of ≥ 2, and the incident ROA for a knee was defined if its KL grade was no more than grade 1 at baseline visit and no less than grade 2 at the follow-up visit. A patient without ROA in both knees at the baseline visit and with ROA in at least one knee at the follow-up visit was viewed as an incident case of ROA in patient level.

Radiographs were read twice by two readers trained at Boston University, and films from Osteoarthritis Initiatives were used as gold standard as well as training material [13]. For each batch of Shunyi OA Study films (*n* = 100), 10 films from the Osteoarthritis Initiatives study were added to test inter-reader reliability. In addition, 10 previously read knee radiographs from the Shunyi OA Study were fed back to the reader to test intra-reader reliability. Weighted kappa statistics for inter-reader and intra-reader reliability were 0.82 and 0.95 respectively.

### Assessment of covariates

Demographic information, such as age, sex, education level, history of knee injury smoking, alcohol consumption, manual occupation, marriage, was collected at baseline using a standard questionnaire. Height was measured twice for each subject, using a wall-mounted stadiometer. Weight was assessed using a balance beam scale with a precision to 0.1 kg. Body mass index (BMI) was calculated as weight in kilograms divided by height in meters squared. A subject with BMI ≤ 25, 25 < BMI ≤ 30, BMI > 30 was defined as normal, overweight and obesity, respectively. History of knee injury was identified by asking subjects “Have you ever suffered an injury in your knees causing a limitation of walking ability for at least one week?” The injured knee side and date of injury were recorded. Range of motion (ROM) and the degree of flexion contracture of the knee joints were assessed by goniometric measurements. A chair stand test was carried out using a 43-cm high, straight back armless chair. A full sit-to-stand and consecutive stand-to-sit cycle was counted as one chair stand, and the total time for 5 cycles was calculated by a well-trained examiner [14]. A 50-foot walk test was carried out by asking the participants to walk in a 25-foot straight footpath and return back to the starting point as fast as possible. The total time was recorded [15].

### Statistical analysis

All data was analyzed by IBM® SPSS® Statistics (Version 24.0) software and was presented as means ± standard deviation (SD). Difference between follow-up group and non-follow-up group was assessed by independent samples *T* test and chi-squared test. Generalized linear model (GLM) was performed to examine the association between factors (including age, sex, BMI, education levels, history of knee injury, chair stand test, 50-foot walk test) and incidence ROA in patient level. ROM, flexion contracture, knee pain, and KL grade at baseline were examined by generalized estimating equation (GEE) in knee level. A two-sided *P* value 0.05 was considered statistically significant.

## Results

### Participants

A total of 1295 residents were recruited at the baseline in 2014, and 967 (74.7%) were followed in 2017. Four hundred ninety-one residents suffered from one or both knee ROA at the baseline visit diagnosed by knee X-ray were excluded from the further incidence analysis. Of the remaining 803 residents, 598 residents received follow-up examination (follow-up rate 74.5%, Table 1). The mean age of following subjects was 59.18 ± 6.57, of which 63.7% were female. The mean BMI was 25.96 (SD = 3.43). The mean knee ROM was 129.67 degrees (SD = 6.60). A total of 85 subjects had knee pain symptoms (14.3%) (Table 1). Among 1196 knees without knee OA at baseline, 776 knees had a KL of 0, and 420 had a KL grade of 1.

**Table 1:** Baseline characteristic of participates

Characteristic	Follow-up	Non-follow-up	*P* value
Total (%)	598 (74.5%)	205 (25.5%)	
**Baseline socio-demographic, BMI, and history of knee injury**			
Age	59.18 ± 6.57	61.48 ± 8.52	<0.01
BMI	25.96 ± 3.43	26.00 ± 3.98	0.283
Gender (%)			
Male	217 (36.3%)	98 (47.8%)	<0.01
Female	381 (63.7%)	107 (52.2%)	
Education (%)			
Primary school	136 (22.7%)	44 (21.5%)	0.705
Middle school or above	462 (77.3%)	161 (78.5%)	
Injury (%)			
Yes	30 (5%)	10 (4.9%)	0.937
No	568 (95%)	195 (95.1%)	
**Baseline physical function of the knee**			
Range of motion in knee level	129.67 ± 6.60	129.70 ± 7.44	0.945
Flexion contracture in knee level	1.23 ± 2.21	1.25 ± 2.24	0.898
50-foot walk test	12.68 ± 2.62	13.48 ± 4.61	0.02
Chair stand test	8.45 ± 2.19	8.87 ± 3.61	0.115
**Baseline knee condition**			
Baseline K/L grade in knee level (%)			
0	776 (64.9%)	248 (60.5%)	0.11
1	420 (35.1%)	162 (39.5%)	
Baseline knee pain (%)			
No pain	513 (85.8%)	185 (90.7%)	0.188
One knee pain	53 (8.9%)	11 (5.4%)	
Both knee pain	32 (5.4%)	8 (3.9%)	

There was significant difference in age, gender, and walk speed tested by 50-foot walk test between follow-up group and non-follow-up group. Older and male residents (mean age 61.48 ± 8.52 years old, female accounted for 52.2%) with poor walk ability tended to lose in the follow-up compared with the subjects who were successfully followed up.

### Incidence of radiographic osteoarthritis

**Fig. 1:**
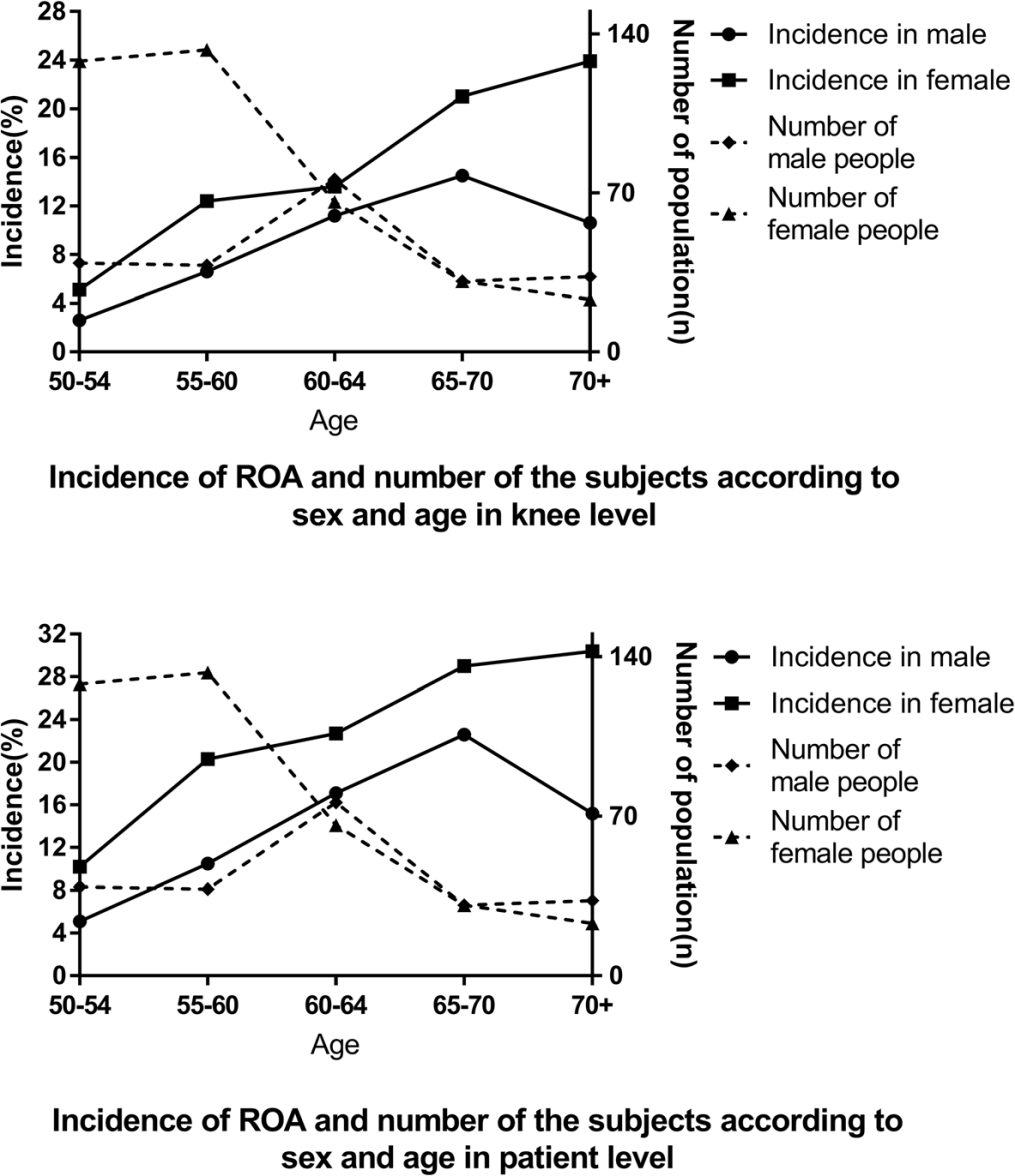
Number of the subjects and incidence of ROA according to sex and age

Incidence was calculated in both knee level and patient level. Five hundred ninety-eight subjects with 1196 knees without radiographic osteoarthritis at baseline were taken into analysis. After 3 years follow-up, 102 subjects with 128 knees developed radiographic osteoarthritis according to the X-ray result. The incidence of ROA was 10.7% (9.2% in male and 11.5% in female) during 3 years (3.6% annually) in knee level and 17.1% (14.3% in male and 18.6% in female) during 3 years (5.7% annually) in patient level. According to the statistical year book of Beijing Shunyi 2017, male villagers constituted 61.87% of the population in Zhaoquanying Town, for which the sex-standardized ROA incidence was 10.1% (3.4% annually) in knee level and 15.9% (5.3% annually) in patient level. The number of the population and incidence of ROA by sex and age are shown in Fig. 1. The incidence was much higher in females than males in all age groups. The incidence of ROA increased with age. However, after the age of 70 the incidence in male decreased and the rising trend of the incidence in females slowed down.

### Risk factors for radiographic osteoarthritis

**Fig. 2:**
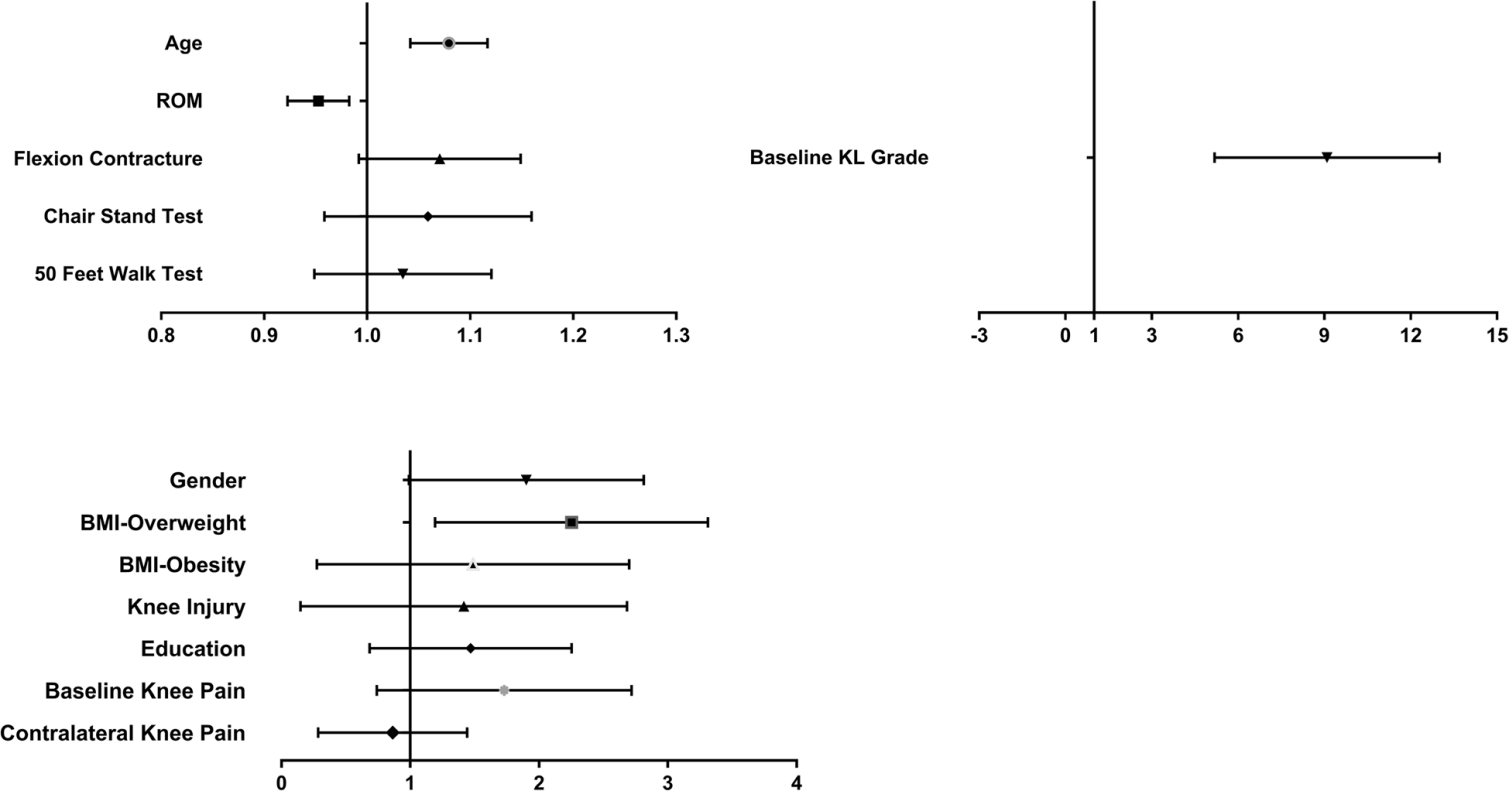
Forest plot for the risk factors analysis of incident radiographic osteoarthritis in model 2

Table 2 and Fig. 2 showed the association between socio-demographic factors, physical function, baseline knee conditions, and incident ROA. In the univariate analysis, advanced age, overweight, less range of motion, and KL grade 1 at baseline were significantly associated with incident ROA. More flexion contracture, knee pain at baseline, poor physical function of lower limb measured by chair stand test tended to be statistically significant. After adjusting for age and gender, advanced age, female, overweight, less ROM, and KL grade 1 at baseline were significantly associated with incident ROA. The significant risk factors (including flexion contracture) were the same after adjusting for age, gender, BMI, education level, and history of injury.

**Table 2:** Association between socio-demographic factors, physical function, baseline knee conditions, and incident ROA

Characteristic	Incident OA (*n* = 102)	Non-incident OA (*n* = 496)	Crude OR (95% CI)	*P* value	Adjusted OR (model 1) (95% CI)	*P* value	Adjusted OR (model 2) (95% CI)	*P* value
**Baseline socio-demographic, BMI, and history of knee injury**								
Age^a^	61.15 ± 6.37	58.77 ± 6.54	1.053 (1.021-1.087)	<0.01	1.067 (1.032-1.102)	<0.01	1.079 (1.042-1.117)	<0.01
Gender^a^								
Male	31 (30.4%)	186 (37.5%)	1.0 (reference)		1.0 (reference)		1.0 (reference)	
Female	71 (69.6%)	310 (62.5%)	1.373 (0.867-2.174)	0.177	1.815 (1.111-2.964)	0.017	1.756 (1.071-2.877)	0.025
BMI^a^								
Normal	35 (34.3%)	224 (45.25%)	1.0 (reference)		1.0 (reference)			
Overweight	60 (58.8%)	225 (45.4%)	1.699 (1.076-2.682)	0.023	2.072 (1.278-3.358)	0.003	2.086 (1.286-3.385)	<0.01
Obesity	7 (6.9%)	47 (9.5%)	0.940 (0.394-2.246)	1.118	0.459 (0.459-2.742)	0.806	1.158 (0.474-2.831)	0.747
History of knee injury^a^								
No	97 (95.1%)	471 (95%)	1.0 (reference)		1.0 (reference)		1.0 (reference)	
Yes	5 (4.9%)	25 (5%)	0.961 (0.359-2.572)	0.937	0.983 (0.364-2.652)	0.973	1.038 (0.381-2.829)	0.942
Education^a^								
Primary school	19 (18.6%)	117 (23.6%)	1.0 (reference)		1.0 (reference)		1.0 (reference)	
Middle school or above	83 (81.4%)	379 (76.4%)	1.351 (0.787-2.319)	0.275	1.320 (0.764-2.283)	0.32	1.329 (0.764-2.313)	0.314
**Baseline physical function of the knee**								
Range of motion^b^	128.27 ± 7.10	129.95 ± 6.46	0.955 (0.930-0.981)	<0.01	0.954 (0.928-0.981)	<0.01	0.952 (0.923-0.983)	<0.01
Flexion contracture^b^	1.46 ± 2.86	1.19 ± 2.15	1.071 (0.999-1.147)	0.052	1.072 (0.998-1.151)	0.058	1.068 (0.993-1.150)	0.078
50-foot walk test^b^	13.14 ± 2.86	12.58 ± 2.57	1.081 (1.003-1.164)	0.041	1.002 (0.941-1.11)	0.601	1.032 (0.950-1.122)	0.452
Chair stand test^a^	8.82 ± 2.13	8.37 ± 2.20	1.087 (0.994-1.188)	0.066	1.056 (0.962-1.159)	0.252	1.056 (0.960-1.161)	0.265
**Baseline knee joint condition in knee level**								
Baseline K/L grade^b^								
0	90 (44.1%)	831 (83.8%)	1.0 (reference)		1.0 (reference)		1.0 (reference)	
1	114 (55.9%)	161 (16.2%)	8.769 (5.905-13.022)	<0.01	8.552 (5.575-13.120)	<0.01	8.527 (5.489-13.246)	<0.01
Baseline knee pain^b^								
No	179 (87.7%)	900 (90.7%)	1.0 (reference)		1.0 (reference)		1.0 (reference)	
Yes	25 (12.3%)	92 (9.3%)	1.646 (0.970-2.793)	0.065	1.560 (0.903-2.694)	0.111	1.541 (0.848-2.800)	0.156
Contralateral knee pain^b^								
No	179 (87.7%)	900 (90.7%)	1.0 (reference)		1.0 (reference)		1.0 (reference)	
Yes	25 (12.3%)	92 (9.3%)	0.960 (0.504-1.831)	0.902	0.770 (0.383-1.546)	0.462	0.735 (0.361-1.496)	0.395

Model 1: Adjusting for age and gender

Model 2: Adjusting for age, gender, BMI, education and history of injury

^a^Tested by Generalized Linear Model in patient level

^b^Tested by Generalized Estimating Equation in knee level

## Discussion

To our knowledge, the current study is one of the first population-based, longitudinal, and prospective cohort studies demonstrating epidemiological characteristics and risk factors for knee OA in an elderly population in China. A total of 1295 residents were recruited at baseline in the year of 2014, and 962 (74.3%) residents were followed in the year of 2017. We found that the incidence of ROA annually was 3.6% in knee level and 5.7% in patient level. Advanced age, overweight, less range of motion at baseline, and KL grade 1 at baseline were relevant risk factors for incident ROA.

The incidence reported in previous studies varied across countries and racial groups. It was reported that the incidence of knee OA was higher among Asian residents than among white population [10, 16, 17]. In the Framingham study with a mean follow-up interval of 8.1 years, the annual incidence of ROA was 1.4% in male and 2.2% in female in patient level [8]. While in Chingford Women’s study, the incidence was 2.3% every year in knee level in the 5 years follow-up period [7]. In the research on osteoarthritis/osteoporosis against disability (ROAD) study in Japan, 6.9% male (2.1% annually) and 11.9% female (3.6% annually) developed incident ROA after 3.3 years from baseline visit [17]. In the Hallym Aging Study (HAS) in Korea, 3.1% in male and 3.7% in female suffered from incident ROA per year [10]. Compared with foreign research results, the incidence in our study was higher.

The higher progression rate of radiographic knee OA in our study might be due to the following reasons. First, our study had a higher follow-up rate (74.5%) compared to previous studies (the Chingford study in the UK 55.9%, the Framingham study in America 60.4%, the ROAD study in Japan 74.4%, the Hallym Aging Study (HAS) in Korea 63.9%). It is generally believed that lower percentage of defaulters can bring less bias to the estimation of the incidence. Patients with severe osteoarthritis, with less walking ability, were much easier to be absent, which decreased the rate of the incidence. Second, the incidence of knee OA may have regional difference. Marita et al. found that the prevalence of knee OA was highest in the Asia Pacific developed region, followed by Oceania and North Africa/Middle East, and that was lowest in South and Southeast Asia. Most data of knee OA coming from developed countries, it was thought that there may be a high incidence in undeveloped and developing countries [18]. Third, our study investigated the population in suburban area, most of whom were farmers bearing heavy workload at their young age, which may raise the knee OA incidence in their elder age [8]. Although the average age of the population was 70.8 years old in Framingham study, 68.7 years old in ROAD study, 71 years old in HAS, which were much higher than that in the present research, we found that the incidence may decline with age in the elderly, especially in people over 70 years old according to our results in Fig. 1 and previous studies [17, 19]. Taking all the above factors into consideration, we can draw the conclusion that the incidence in Beijing suburban area was higher.

Our study also found that advanced age, female, overweight, less range of motion, and KL grade 1 at baseline were significantly associated with incident ROA.

Advanced age, female, and higher BMI have long been viewed as main risk factors of ROA [20, 21]. Recently, a meta-analysis conducted by Silverwood showed that obesity, female gender, and previous knee injury were the main factors associated with onset of knee pain [20]. In most studies, incidence of ROA had positive correlation with age. However, our study found that the rate of increase in the incidence slowed down or even decreased in elderly people over 70 years old. There were few studies which also demonstrated a continuously increasing risk of knee and hip OA after the age of 40 for both genders, peaking at ages 75-80, and falling back over 80 [19].

Less ROM is also a significant risk factor for incident knee osteoarthritis. Akinobu Nishimura firstly reported the relationship between ROM and ROA [11]. They found that a low knee ROM was the only risk factor for progressive knee OA. In particular, OA progressed more quickly among participants with a knee ROM of < 120° than among those with a knee ROM of > 120°.In another study, Reijman et al. reported that restricting hip flexion by > 20% is a risk factor of progressive hip OA [22]. The same mechanism involved in hip OA might also function in knee OA because the knees are also weight-bearing joints. However, it is not entirely clear how low knee ROM causes knee OA to progress, and the relevant mechanism needs further study. Special attention should be paid to the patients with low ROM, and effective measures should be taken to prevent knee OA progression.

Some investigators claimed that subjects with KL grade 1 at baseline should be treated differently because those doubtful osteophytes highly predict OA in the near future [23, 24]. In this study, we found that KL grade 1 at baseline was strongly associated with radiograph progression of knee OA. In 2014, establishing the ROA prognostic model, Kerkhof found that adding the knee baseline KL grade significantly increased the AUC, which showed that knee baseline KL grade was a very strong predictor of future knee OA [24]. A KL grade of 1 was by far the best predictor of future knee OA, and even stronger than age, gender, and BMI. The most likely explanation for the strong predictive value of these minor radiographic changes is that this score is an indicator of early-stage OA and suggests that some structural damage has already occurred. Therefore, the probability of progression to a definite knee OA (KL ≥ 2) is higher compared with people without any signs of OA on a radiograph.

Knee joint osteoarthritis is often accompanied by flexion contracture (FC). Our study found that flexion contracture tended to be statistically significant in both univariate and multivariate analysis, which showed that flexion contracture might be a risk factor for knee osteoarthritis incidence. Using the OAI data, Campbell found that knee FC was a risk factor for radiographic OA incidence, joint space narrowing, worse clinical outcomes, radiographic progression, and the need for early TKA [25]. Biomechanically, knee FC limits the cartilage surface concentrating loads over a smaller articular area, which potentially accelerates the cartilage damage due to the increasing local hydrostatic pressures, leading to chondrocyte apoptosis and further cartilage degeneration.

Frailty and low limb physical function has been proved to be associated with prevalent ROA [26]. Chair stand test and 50-foot walk were recommended by the Osteoarthritis Research Society International as the core set of performance-based measures. However, few studies have examined the association between low limb physical function and incident ROA. In our study, we found that the result of chair stand test, 50-foot walk were not statistically significant after adjusting basic demographic information.

A study conducted in symptomatic population showed that a 3-year-cumulative incidence of ROA with pain was 21.7%, which was pretty higher compared with the study mentioned above conducted in normal population, proving the relationship of pain and ROA [27]. Baseline knee pain has been proved to be related with a reduction in thigh muscles strength, and it is well accepted that strong thigh muscles are able to reduce the incidence of OA [28, 29]. Some researchers believe that baseline knee pain can be a promotive factor for the disease. However, a previous prospective study concluded that widespread pain including pain on either knee joint was not significantly associated with either incident unilateral or bilateral ROA [30]. In our study, we found that baseline knee pain was not significantly associated with incidence of ROA. The relationship between the two remains a controversial issue and still needs further study.

Injury in young age has been proved to be a risk factor related to osteoarthritis except for non-specific injury [31, 32]. In the present study, there was no significant correlation between injury history and incidence of ROA. This was inconsistent with findings in previous studies. Sayre et al. reported that there was no evidence that history of non-specific injury (NSI) affects knee ROA incidence and progression in a population with knee pain, adjusting for SI, age, sex, BMI, KL grade, and follow-up time, while specific injury relating to cruciate ligament tear, collateral ligament tear, meniscal tear, or patellar injury was associated with increased incidence of ROA [31]. If confirmed, the null finding means that people who suffer non-specific injuries to the knee (no apparent damage to ligaments, meniscus or patella) need not worry about a subsequent, long-term, increased risk of radiographic OA incidence/progression, even when the NSI is severe enough to require a walking aid for at least 1 week. This result is important, as an increasing popularity of physical exercise may lead to more knee injuries, both specific and nonspecific. In addition, those who are overweight and are asked to begin exercise programs (which ought to reduce rates of ROA) represent another growing group at risk for knee injury.

With regard to low education level, it is widespread accepted to be associated with prevalence and progression of osteoarthritis [33, 34]. According to our study and previous foreign study, it may account for ROA prevalence and progression instead of incidence, for the higher education attainment may be more helpful for self-management in the early stage of osteoarthritis patients.

### Strengths and limitations

Our study had strengths and limitations. First, to our knowledge, our study was one of the first prospective study to investigate the incidence of knee ROA in China. We found that the incidence in Beijing suburban area was higher than those in other published regions. Second, we identified risk factors in demographic information, physical examination, physical function test, and imaging, which may offer more evidence-based policy-making for prevention and treatment of ROA. We found that advanced age, overweight, less range of motion, and KL grade 1 at baseline were relevant risk factors for incident ROA. However, despite its prospective design, 3 years is a rather short time to evaluate the progression of OA. Our study contains a relatively small sample size and a high percentage of female samples, which may cause difficulties in risk factors analysis and overestimate the incidence rate of OA. Some subjects with poor walk ability were more likely to be lost in the follow-up, which may bring bias for evaluation for both rate and factors. All the subjects came from Zhaoquanying, a small town in the suburban area around Beijing, may reduce the representativeness of the sample.

## Conclusion

The incidence of knee ROA in Chinese suburban area was high. Advanced age, female, overweight, less range of motion, and KL grade 1 at baseline were associated with an increased risk of incident ROA.

## Data Availability

Data will be available upon request by the first author LZ.

## Abbreviations

OA: Osteoarthritis
ROA: Radiographic osteoarthritis
KL: Kellgren and Lawrence
OAI: The Osteoarthritis Initiative
BMI: Body mass index
SD: Standard deviation
ROM: Range of motion
NSI: Non-specific injury
FC: Flexion contracture
